# Green walnut husk extracts Proliferation and Migration in Gastric Cancer

**DOI:** 10.7150/jca.57270

**Published:** 2022-01-09

**Authors:** Jiarong Zhang, Jinli Zhang, Chunbo Zhao, Hong Sui, Chun feng Li, Lili Zhong, Qingxin Zhou, Yuxian Bai, Shijia An, Xiaoxue Du, Xiaoli Wei, Lei Liu

**Affiliations:** 1 Department of Internal Medicine, Harbin Medical University Cancer Hospital, Haping Road 150 of Nangang District, Harbin, Heilongjiang Province, 150081, China.; 2 Department of Radiotherapy, Harbin Medical University Cancer Hospital, Haping Road 150 of Nangang District, Harbin, Heilongjiang Province, 150081, China.; 3 Gastrointestinal Surgical Ward, Harbin Medical University Cancer Hospital, Haping Road 150, Harbin, Heilongjiang, 150081, China.; 4 Department of Pathology, The Affiliated First Hospital, Heilongjiang University of Chinses Medicine, Heping Road 24 of Xiangfang District, Harbin, Heilongjiang Province, 150081, China.; 5 Department of Medical Oncology, Fujin Central Hospital, Heilongjiang Province, 156100, China.

**Keywords:** The green walnut husk, Anticancer, Gastric cancer, Cell apoptosis

## Abstract

**Background:** In the past few decades, natural products have become an increasingly important source of potential anti-cancer agents. The green walnut husk(GWH) extracts have been reported to inhibit multiple tumor cells and might be a promising chemopreventive agent in human neoplasia. However, it is not clear whether GWH extracts inhibit gastric cancer.

**Methods:** Proliferation, invasion, and migration of gastric cancer cells were assessed by the CCK-8, wound-healing, and Transwell assay. The apoptotic rate was detected by flow cytometry(FCM). The expressions of Bcl-2, Bax, and Caspase-3 proteins were examined by Western blot. Moreover, the growth of gastric cancer cells was assessed using orthotopic xenograft models, and related proteins expressions were evaluated using immunohistochemistry. Finally, the Gene expression profile of gastric cancer treated with GWH extracts was evaluated by using High-throughput RNA sequencing(RNA-seq).

**Results:** GWH extracts effectively inhibited gastric cancer cell growth *in vitro* and *in vivo*. *In vivo*, GWH extracts inhibited the survival of gastric cancer cells in a dose and time-dependent manner, inhibited the migration and invasion of gastric cancer cells, regulated the expressions of apoptosis-related proteins, and induced apoptosis of gastric cancer cells. *In vitro*, GWH extracts inhibited the growth of mouse xenografted tumors. A total of differentially expressed genes, of which 41 genes were up-regulated, and 610 genes were down-regulated, were identified by RNA-seq. GO, and KEGG analysis showed that these differentially expressed genes might be related to the mechanism of the anti-gastric cancer effect of GWH extracts.

**Conclusion:** GWH extracts played an anti-gastric cancer effect by inducing apoptosis and inhibiting invasion. Secondly, the differential expression of many genes, multiple signal pathways, and metabolic pathways in gastric cancer play an essential role in the anti-gastric cancer effect of GWH extracts. The results suggested that GWH extracts are expected to be a low toxic drug for the treatment of gastric cancer in the future.

## Introduction

Globally, gastric cancer (GC) is the fifth most frequently diagnosed cancer and the third leading cause of cancer death 1. Due to its often advanced stage at diagnosis, mortality from gastric cancer is high; gastric cancer has become one of the most aggressive and heterogeneous diseases in the world 2. Chemotherapy is the main treatment option for patients with advanced or metastatic GC 3. And patients with advanced gastric cancer treated with combination chemotherapy have a median overall survival of around one year 4. Drug resistance of tumor cells is one of the main causes of chemotherapy failure 5, due to the substantial heterogeneity of gastric cancer will lead to acquired or primary drug resistance 6, the rapid development of drug resistance, and serious side effects limiting traditional chemotherapy drugs. Therefore, it is an urgent task to explore new therapeutic agents or a new combination of agents to improve the treatment of gastric cancer. At present, many studies have shown that a variety of traditional Chinese medicine can play an anti-tumor role 7-9.

The walnut (Juglans regia L.) is a critical deciduous tree species found principally in temperate areas worldwide. It is cultivated throughout eastern Asia, southern Europe, northern Africa, the United States, western South America, and India. According to statistics, world production of whole walnut (with shell) was around 1.5 × 10^6^ t in 2008 10. In 2017, the global walnut planting area was 109,7700 hm^2^, and walnut planting area in China accounted for 44.63%, mainly distributed in northeast, north, northwest, central China, and other regions. China is the leading world producer. The walnut has many medicinal parts, such as roots, barks, leaves, stem barks, and green husks. The green walnut husk (Chinese name: Qing Long Yi) was used in folk medicine because it shows antioxidant 11, antitumor 12-14, and antibacterial properties 15. The green walnut husk extracts are ethanol extracts from the immature exocarp of the walnut. Naphthoquinones were the main anti-tumor constituent of the green husks of walnut, especially juglone 16. Modern pharmacological studies have shown that Juglone, the leading organic extract of the green walnut husk, can play an anti-tumor role by inducing apoptosis, inhibiting proliferation, migration, invasion, and cell cycle arrest, suppressing epithelial-mesenchymal transition (EMT) 17, eliminating the accumulation of Myeloid-derived suppressor cells (MDSCs) 18, and other mechanisms. Previous research indicated that the green walnut husk extracts inhibited the growth of many tumor cells, including liver cancer 14, breast cancer 19, and colon cancer 20. However, it is not clear whether the green walnut husk extracts affect gastric cancer's anti-cancer activity. The purpose of this study is to research on the anti-gastric cancer effect of the green walnut husk extracts.

In this study, firstly, we investigated the effects of the green walnut husk extracts on apoptosis and invasion of human gastric cancer cells *in vitro* and *in vivo*. Secondly, we used RNA-seq to study the expression profile of differentially expressed genes in gastric cancer tissues after the green walnut husk extracts treatment. The purpose of this study was to reveal the anti-gastric cancer effect of the green walnut husk extracts and used RNA-seq and bioinformatics techniques to find their main metabolic pathways and signal pathways related to different expressed genes and explored their clinical application value. This study could provide an experimental basis for exploring the molecular mechanisms of the green walnut husk extracts as an anti-tumor agent.

## Methods and materials

### The ethanol extracts of the green walnut husk preparation

The ethanol extracts of the green walnut husk preparation. The green walnut husks of Juglans Mandshurica Maxim were collected from Chang bai Mountain (Jilin, China), identified by Professor Libo Wang. The specimen (No. 20180902) has been deposited in the College of Pharmacy, Harbin Medical University. The dried green walnut husks (1.0 kg) have been extracted with 95% ethanol (1.0 L) 4 times and evaporated under vacuum to give crude extracts (60.0 g). The ethanol extracts of the green walnut husks were abbreviated to GWH extracts.

### Cell culture

The human gastric cancer cell line SCG7901 (Laboratory of Medical Genetics, Department of Biology, Harbin Medical University, Harbin, China) was grown in RPMI 1640 medium containing 10% heat-inactivated fetal bovine serum (FBS) and 100 U/ml penicillin/streptomycin. Cells were maintained in a humidified atmosphere of 5% CO_2_ at 37°C. Once cells reached confluence, they were subcultured by using trypsin digestion.

### Cell viability and colony formation assay

Cell proliferation was measured with CCK-8 according to the manufacturer's instructions. Briefly, SCG7901 cells were seeded at a density of 3×10^3^ cells/well in a 96-well plate and cultured for 24 h. GWH extracts were then added to the wells with final concentrations of 10, 20, 30, 50,100, and 200 μg/mL and incubated for 6, 12, 24, and 48 h. Before detecting the absorbance, 10 µl CCK-8 was added to each well and incubated for an additional 1.5 h at 37˚C. Finally, the absorbance at 450 nm was measured using a Multiskan Spectrum microplate reader (Thermo Fisher Scientific, Waltham, MA, USA).

In the colony formation assay, the gastric cancer cells (600-800 cells/well) were inoculated in 6-well plates and cultured for 24 h. Then GWH extracts (100 μg/mL) were added and incubated for eight days. After fixed with 4% paraformaldehyde and stained with crystal violet solution, the cells were observed and counted under the microscope.

### Apoptosis morphologic changes analysis

The gastric cancer cells (1-2×10^5^ cells/well) were inoculated in 6-well plates and cultured for 24 h. Then added GWH extracts (100 μg/mL) with 2 ml and incubated 24 h, then the mixture of AO/EB (100 μg/mL AO and 100 μg/mL EB mixed in the ratio of 1: 1) was added at room temperature for 5 min in the dark. Then cells were observed under the fluorescence microscope.

### Cell migration and invasion

Cells were seeded in six-well plates with a complete DMEM medium to analyze wound healing. After 48 h, the cell monolayer was scratched with a plastic pipette tip. Then cells were rinsed with PBS and cultured with serum-free DMEM for 24 h. The wound closure was observed and photographed under a microscope. For the Transwell assay, 8- μm pore size chambers (Corning, NY, USA) were used with an insert coated with Matrigel (BD Bioscience). After 24 hours of GWH extracts treatment, one × 10^5^ cells in 200 μL serum-free medium were added to the upper chamber. The lower section was filled with 500 μL 10% FBS DMEM. Cells remaining on the upper surface of the membrane were removed after 24 h of incubation, whereas cells that had invaded through the membrane were fixed with 100% methanol for 15 min, stained with 0.1% crystal violet for 20 min. Cells were examined and photographed under a phase-contrast microscope (Olympus, Japan).

### Flow cytometry analyses for apoptosis

Cells were cultured with GWH extracts (100 μg/mL) for 24 h. For apoptosis analysis, GWH extracts-treated cells and their control cells were collected, washed, and mixed with five μL FITC conjugated annexin-V and five μL propidium iodide (PI) for 15 min at room temperature in the dark. Then, cells were resuspended with PBS for flow cytometry analyses (BD Bioscience, USA).

### Tumor xenograft

According to the Animal Ethics Procedures and approved by the Harbin Medical University Animal Ethics Committee, all animal experiments were performed according to the Animal Ethics Procedures. 30 male BALB/c nude mice (SPF level, Shanghai SLAC Laboratory Animal Co., Ltd) were randomized into five groups: control group, positive control group (S1 50 mg/kg), high (GWH extracts 500 mg/kg), middle (GWH extracts 200 mg/kg) and low (GWH extracts 100 mg/kg) dose groups, six animals in each group. Then SCG7901 cells were injected subcutaneously into nude mice with the number of 5×10^6^/ mice. Twenty-four hours after the injection, drugs were administered by oral gavage once a day for two weeks. Tumor growth was observed and recorded for 20 days. After treatment, the mice were sacrificed, and the tumors were removed, weighed, and fixed for use in immunohistochemical experiments.

### Western blot analysis

SCG7901 cells (3×10^5^) seeded in 6-well plates were incubated with GWH extracts (100 μg/mL) for 24 h and incubated specific primary antibodies overnight without light, such as β-catenin (Danvers, MA, USA), β-actin (BD Biosciences, Bedford, MA, USA), Bal-2 (Wanlei, China, wl01556), Bax (Wanlei, China, wl01637), Caspase-3 (Wanlei, China, wl01992), Rb (Abcam, Cambridge, MA, USA, ab25901), and p53 (Wanlei, China, wl03426). After flushed, the second antibody labeled by fluorescent labeling was incubated without light for 1 hour. Western blot analysis was performed using these antibodies.

### Histopathology and immunohistochemistry

The tumor specimens of GWH extracts before treatment (control group) and after GWH extracts treatment (high dose group) were fixed in 10% formalin buffer for 24 h and embedded in paraffin and sliced. Ki67 antibody, E-Cadherin antibody, MLH1 antibody, MSH2 antibody, MSH6 antibody, PMS2 antibody, and Vimentin antibody diluted with PBS buffer were added to the sample. Then the fluorescent secondary antibodies labeled with FITC were added respectively. Then incubated at room temperature without light for 20 minutes, rinsed five times with PBS buffer, used DAB developer, re-dyed with hematoxylin dye solution for 2 min, rinsed with PBS buffer, and sealed with neutral gum. Images were captured under a light microscope (100×, 400× Leica, Germany).

### RNA-seq analysis

They were divided into experimental groups and control groups for RNA-seq. The experimental group was the tumor tissue of the high-dose group of nude mice, and the control group was the tumor tissue of the control group. And the total RNA was extracted by Trizol (Invitrogen, Carlsbad, CA, USA). The yield and quality of RNA were assessed using Nanodrop 8000, A260/280 ratio 1.8-2.0. RNA integrity was analyzed with Agilent 2100 Bioanalyzer (Agilent Technologies, Santa Clara, CA, USA). In the following experiments, the total RNA samples were used to meet the following requirements: RNA integrity number (RIN) ≥ 7.0 and a 28S: 18S ratio ≥ of 1.5. The preparation of high-throughput RNA sequencing and deep sequencing were performed by Shenzhen Huada Genome Co., Ltd. (Shenzhen, China). The specific methods were as follows, using Ribo-Zero Magnetic Gold Kit (Illumina, USA) enriches mRNA with polyA tail, total RNAs were subjected to ribosomal RNA (rRNA) removal using the RNaseH (Epicentre Technologies, Madison, WI, USA). The obtained RNA is segmented, and the RNA fragment is used as the template, the first-strand cDNA was synthesized from the RNA fragments by reverse transcriptase and random hexamer primers, then synthesized the second strand of cDNA to form dsDNA. The end of the cDNA fragment was subjected to an end repair process which included the addition of a single "A" base, add 5'- terminal phosphorylation, followed by ligation of the adapters. To amplify the library DNA, the qRT-PCR technique was used to purify and enrich the library. Then, the libraries were qualified by Agilent 2100 and quantified using the KAPA Library Quantification kit (KAPA Biosystems, South Africa). Finally, we used the BGISEQ-500 platform for sequencing.

### Bioinformatic analyses

Bioinformatics analysis used SOAPnuke, a filtering software independently developed by Shenzhen Huada Genome Co., Ltd., to filter low-quality data, and the filtered data are saved in FASTQ format 21. The reference genomes were compared by HISAT (Hierarchical Indexing for Spliced Alignment of Transcripts) software 22, and the reference genes were compared by Bowtie2 software 23, 24. The clean reads with high quality were then aligned to the human reference genome (GRCh38/hg38) using Tophat2 software (v2.0.13) 25 with default parameters. According to the results of differential gene detection, the Pheatmap package of R software was used to analyze the differential expression of genes. We classified the differential genes into functional classification and biological pathway classification. The Pyper function of R software was used for Gene Ontology (GO), and Kyoto Encyclopedia of Genes and Genomes (KEGG) pathway enrichment analysis was calculating the pvalue, then the pvalue was corrected by FDR. Usually, Qvalue ≤ 0.05 was regarded as significant enrichment. The purpose of GO and KEGG enrichment analysis was to understand the function and interaction between differentially expressed genes.

### qRT-PCR validation

To verify the reliability of RNA-seq, qRT-PCR was used to detect the expression levels of different genes. SCG7901 cells were exposed to a particular concentration of the green walnut husk extracts (100 μg/mL) for 24 h, and the total RNA was extracted by Trizol (Invitgen, Carlsad, CA, USA). Reverse transcription system was performed in a 20 μl reaction volume, including four μl five × RT buffer, 1μl gDNA remover, 0.5 μgtotal RNA, and 14.5 μl with Nuclease-Free Water, and the reverse transcription reaction was carried out to produce cDNA. qRT-PCR was performed in a 20 μl reaction volume, including ten μl SYBR hybrid system, one μl upstream primer, one μl downstream primer, one μl cDNA, and seven μl Nuclease-Free Water. β-actin was used as a reference. The PCR board was placed in the Roche 96 PCR instrument, and the protocol was initiated at 94°C for 2min, followed by 94°C(20 sec), 60°C(30 sec), and 72°C (30 sec) for a total of 40 cycles, then annealed at 72 ℃ for 5min. The relative expression of RNAs was calculated by the 2-ΔΔCt method. Student's t-tests were performed, and results were considered to show a significant difference when P<0.05. All reactions were performed in triplicate.

### Statistical analysis

Statistical analyses were performed using SPSS 23.0, and figures were produced using GraphPad Prism 7.0. Comparisons between groups were analyzed by Student's T-test, Bonferroni's Multiple tests, and One-way analysis of variance (one-way ANOVA), as appropriate. At least three independent experiments were conducted. A p-value <0.05 was considered statistically.

## Results

### Viability of gastric cancer cell line treated with GWH extracts

**Figure 1:**
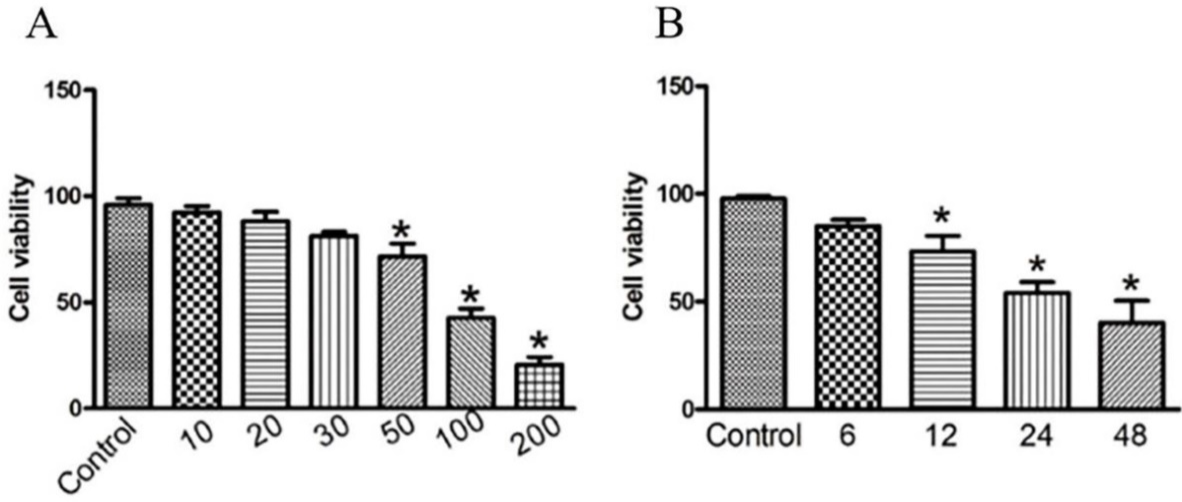
**GWH extracts inhibited cell viability in human gastric cancer cells.** SCG7901 cells were treated with various concentrations of GWH extracts (10,20,30, 50, 100 and 200 μg/mL) for 6 h, 12 h, 24 h and 48 h incubation. Cell proliferation was measured with CCK-8 according to the manufacturer's instructions. Based on the results from the CCK-8 test, GWH extracts at a dose of 100 μg/mL that reduced about 50% cell viability were chosen to use in subsequent experiments. Data are presented as mean ± SD of three independent experiments. *P < 0.05; **P < 0.01 compared with the control.

To investigate the anti-cancer activity of GWH extracts on gastric cancer, we first examined the effect of GWH extracts on the proliferation of SCG7901 cells using the CCK-8 assay, colony formation test. As shown in Figure 1(A), the viability of the cells decreased evidently as the concentrations of GWH extracts increased from 10 to 200 μg/mL. As shown in Figure 1(B), when cultured with GWH extracts for 6, 12, 24, and 48 hours, respectively, we found that the longer the culture time, the more pronounced the decrease of cell survival rate. GWH extracts also showed the antiproliferative effect on SCG7901 cells in a time-dependent manner compared with the control. The above data demonstrated that GWH extracts suppressed human gastric cell proliferation in a concentration- and time-dependent manner. Based on the results from the CCK-8 test, GWH extracts at a dose of 100 μg/mL that reduced about 50% cell viability were chosen to use in subsequent experiments.

### Effects of GWH extracts on the migration and invasion of SCG7901 cells

**Figure 2:**
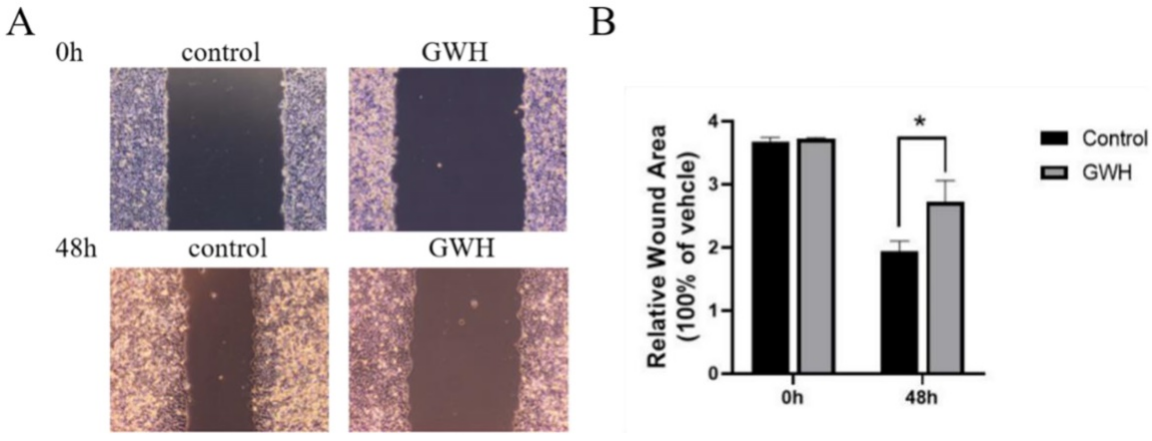
** Effects of GWH extracts on the migration of SCG7901.** After 48 hours of treatment with GWH extracts, the scratches of SCG7901 cells still did not heal, while the scratches of untreated SCG7901 cells in the control group healed gradually after 48 hours of culture compared with 0 hours. Data are presented as mean ± SD of three independent experiments. *P < 0.05; **P < 0.01 compared with the control.

**Figure 3:**
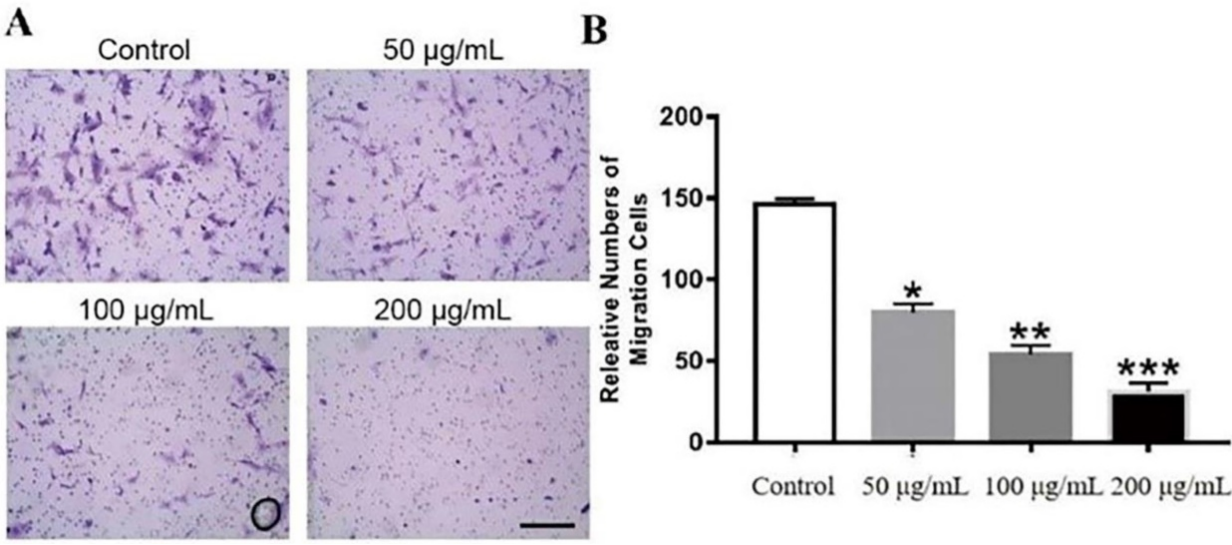
** Effects of GWH extracts on the invasion of SCG7901**. In Transwell assay, the invasive ability of SCG7901 cells treated with 100 μg/mL GWH extracts for 48 h decreased significantly. Data are presented as mean ± SD of three independent experiments. *P < 0.05; **P < 0.01; ***P < 0.001 compared with the control.

The effects of GWH extracts on the migration and invasion of SCG7901 cells were detected by wound healing test and Transwell migration test. As shown in Figure 2, after being treated with GWH extracts for 48 h, the scratches of cells in the SCG7901 group were still not healed after 48 h, while those in the control group disappeared after 48 h of culture. The results of the scratch experiment confirmed that GWH extracts could inhibit the migration of SCG7901 cells. The invasive ability of SCG7901 cells treated with 100 μg/mL GWH extracts for 48 h was detected by the Transwell chamber (Fig. 3). The results showed that 100μg/mL GWH extracts could significantly inhibit the invasive ability of SCG7901 cells. The results confirmed that GWH extracts could inhibit the invasion and migration of human gastric cancer cells.

### GWH extracts decreased the ability of colony formation assay

**Figure 4:**
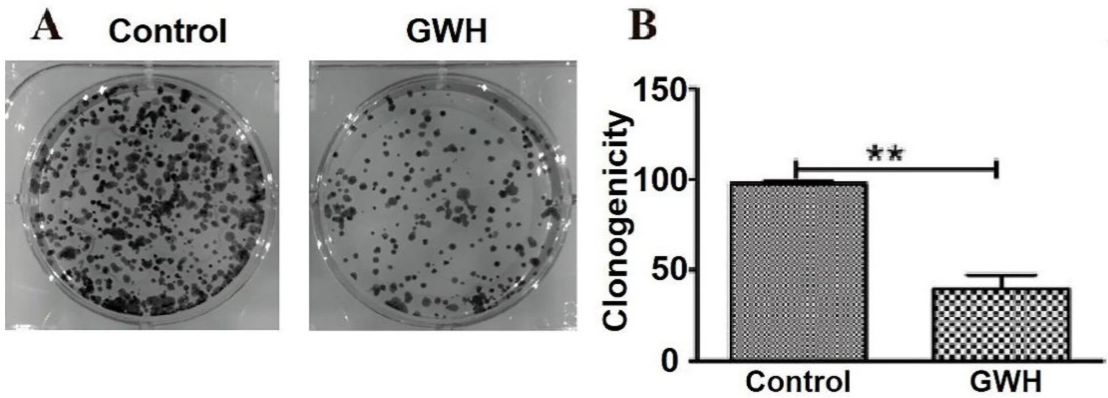
** GWH extracts impacted the colony formation ability of SCG7901.** Compared with the control group, the number of colonies formed by SCG7901 cells decreased significantly after treated with GWH extracts for 24 h. The result showed that GWH extracts decreased the colony forming ability of SCG7901 cells. Data are presented as mean ± SD of three independent experiments. *P < 0.05; **P < 0.01 compared with the control.

Compared with the control group in the colony formation assay, the colony formation of SCG7901 cells decreased significantly after treatment with GWH extracts. The results showed that GWH extracts reduced considerably the colony-forming ability of SCG7901 cells (Fig. 4).

### GWH extracts induced apoptosis of SCG7901 cells

**Figure 5:**
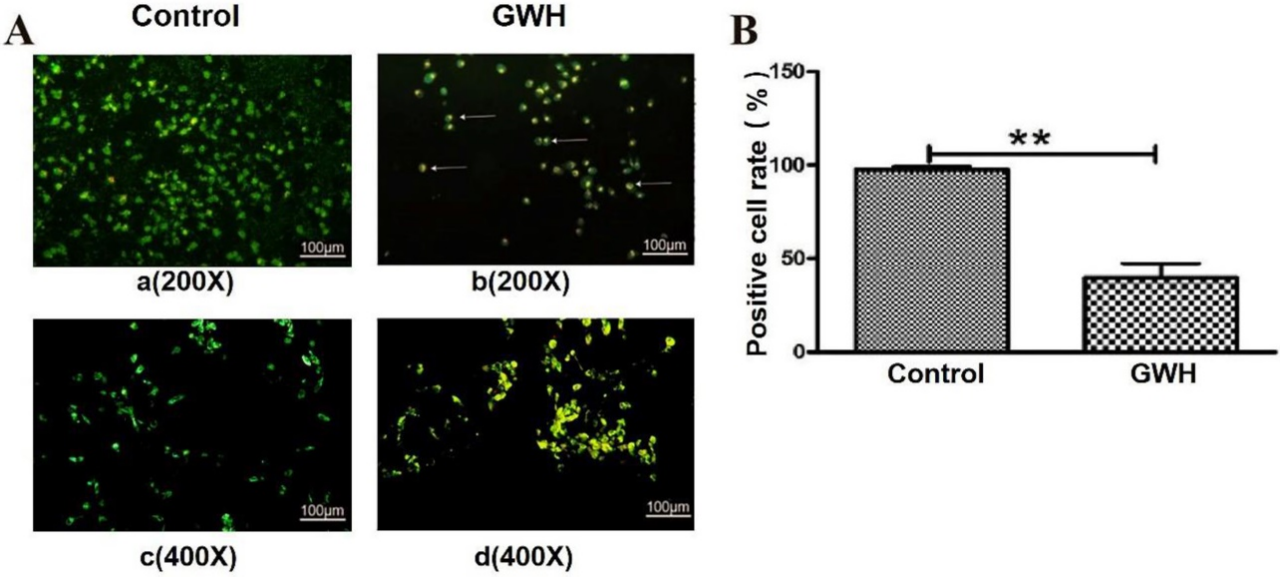
** Morphological changes of SCG7901 cells nuclei after treatment with GWH extracts.** AO/EB fluorescent staining was used to assess cell morphological differences. Under fluorescence microscope (a. b×200, scale bar = 100 µm; c.d×400, scale bar = 100 µm): GWH extracts (100 μg/mL) treated SCG7901 cells appeared as orange-yellow fluorescence with apoptotic nuclear morphological changes at 24 h. These changes including chromatin marginalization, DNA condensation and fragmentation and formation of apoptotic bodies. In untreated cells, there were no morphological changes; nuclei fluoresced as faint green which was homogenous. Data are presented as mean ± SD of three independent experiments. *P < 0.05; **P < 0.01 compared with the control.

**Figure 6:**
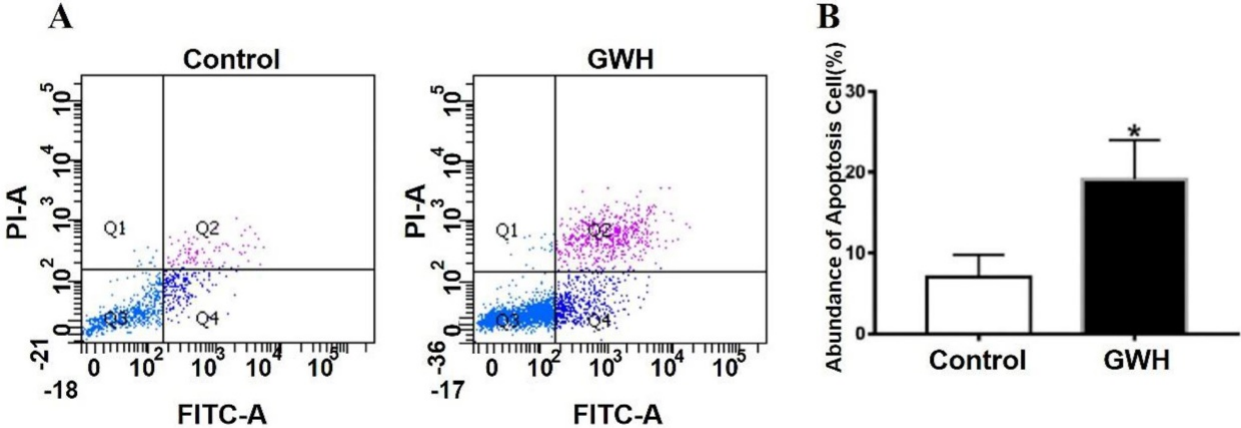
**GWH extracts induced apoptosis of SCG7901 cells.** GWH extracts (100 μg/mL) were co-cultured with SCG7901 cells for 24 h, collected from the cells treated with GWH extracts and its control cells, washed and mixed with 5 μL FITC conjugated annexin-V and 5 μ L propidium iodide (PI) for 15 min at room temperature in the dark, the cells were analyzed using flow cytometry. It is a two-parameter histogram, in which each point represents a cell. Compared with the control group, the number of apoptosis cells increased significantly after treatment with GWH extracts for 24 hours. These data clearly showed that GWH extracts treatment may significantly induced apoptosis of SCG7901 cells. Data are presented as mean ± SD of three independent experiments. *P < 0.05 compared with the control.

The effect of GWH extracts on the nuclear morphology of SCG7901 cells was observed by AO/EB fluorescent staining. AO can enter living cells and emit green fluorescence, but EB only enters apoptotic cells and emits orange-yellow fluorescence. After treatment with GWH extracts (100 μg/mL) for 24 h, SCG7901 cells were accompanied by nuclear morphological changes, including chromatin marginalization, DNA condensation, fragmentation, and apoptotic body formation. In untreated cells, there were no nuclear morphological changes, and the nucleus showed uniform light green fluorescence (Fig. 5). To determine the apoptosis-inducing effect of GWH extracts on SCG7901 cells, Flow cytometry was used to detect the impact of GWH extracts (100 μg/mL) for 24 h. As shown in Figure 6, compared with the control group, the proportion of living cells decreased significantly, and apoptotic cells' proportion increased after treatment with GWH extracts. The results showed that GWH extracts could induce substantially apoptosis of SCG7901 cells.

### Effect of GWH extracts on the expression of apoptosis-related proteins in SCG7901 cells

**Figure 7:**
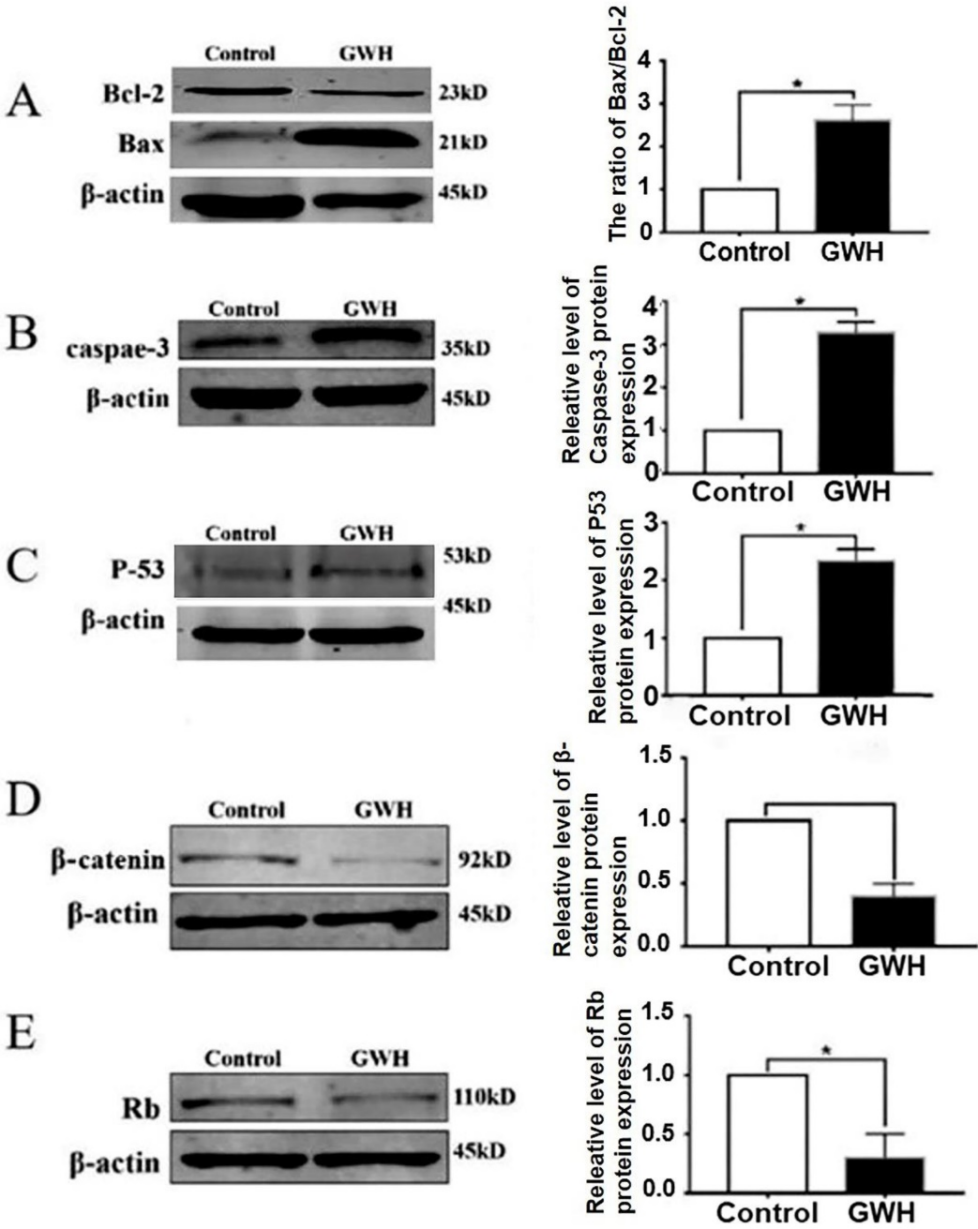
** Western blot anssay detected the effect of GWH extracts on the expression of apoptosis regulatory protein.** Western blot assay revealed the expression of Bcl-2, Bax, β-catenin, Rb, p53 and Caspase-3 proteins in SCG7901 cells treated with GWH extracts for 24 h. β-actin was used as an endogenous reference. Histogram represents the statistical analysis of the relative expression level of Bcl-2/Bax, β-catenin, Rb, p53 and Caspase-3. *P < 0.05 compared with the control.

Next, we studied the possible mechanism of the anti-cancer effect of GWH extracts. Apoptosis is a landmark event for all cancers. Therefore, the impact of GWH extracts on apoptosis regulatory proteins Bax, Bcl-2, Rb, and p53 were evaluated in this study. The expression of cell cycle-related proteins was detected by the Western blotting method (Fig. 7). β-actin was used as a reference. Figure 7 showed that GWH extracts could significantly up-regulate the expression of Bax and down-regulate the expression of Bcl-2. Bcl-2 was a prominent member of the mitochondrial apoptosis pathway. In addition, GWH extracts down-regulated the expression of the β-catenin protein while up-regulated the expression of caspase3, Rb, and p53 protein. The above data suggested that GWH extracts may promote the apoptosis of SCG7901 cells by regulating apoptosis-related proteins.

### GWH extracts inhibited tumor growth in a mouse model of xenograft tumors

**Figure 8:**
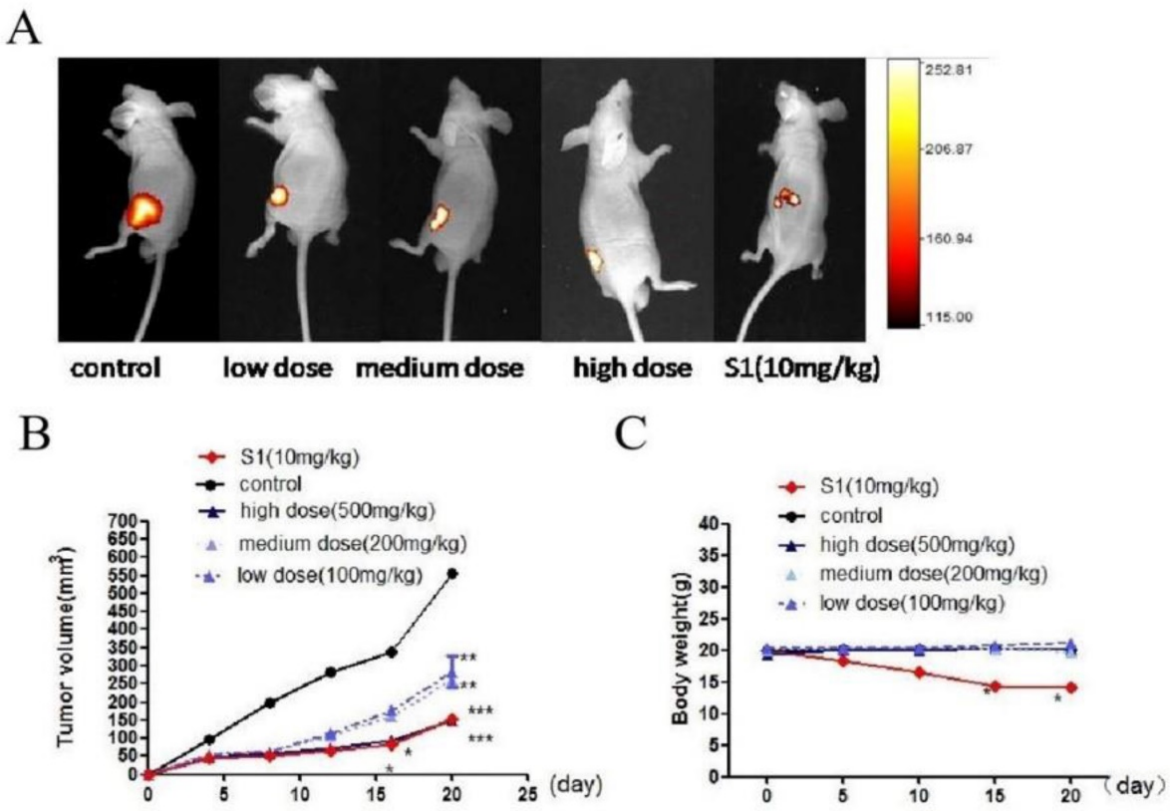
** Effect of GWH extracts on xenografted tumor in mice.** GWH extracts significantly inhibited the growth of tumor xenografts *in vivo*. SCG-7901 tumor cells were inoculated subcutaneously in the BALB/c nude mice. After 24 hours of inoculation, oral administration, GWH extracts were divided into low, middle and high dose groups (100,200,500 mg/kg/d), and the control group and positive control group (S1 10 mg/kg) were set up, once a day for 2 weeks. The tumor growth was observed and recorded. After treatment, the mice were killed. (A)Representative image of human gastric cancer xenograft from the control group, the positive control group and GWH extracts-treated groups. (B)Tumor volume. (C)Mouse weight. *P < 0.05; **P < 0.01; ***P < 0.001 compared with the control.

We used BALB/c nude mouse model to detect the effect of GWH extracts on tumor growth. One day after injection of tumor cells, nude mice were treated with GWH extracts (100, 200, 500 mg/kg) once a day for 14 days, and the tumor growth was monitored for up to 4 weeks. S1 group was used as a positive control compound. From the 15th day after administration, there was a significant difference in tumor size between the drug treatment group and the excipient control group, and the high dose of GWH extracts had the best effect (Fig. 8A, Fig. 8B). However, there was no significant difference in the average body weight between the GWH extracts group and the control group, but the bodyweight of the S1 group decreased significantly (Fig. 8C). The three doses of GWH extracts had an apparent inhibitory effect on tumor growth, consistent with that of S1. These results showed that GWH extracts could also inhibit the growth of gastric cancer *in vivo*, and the bodyweight of mice did not decrease significantly.

**Figure 9:**
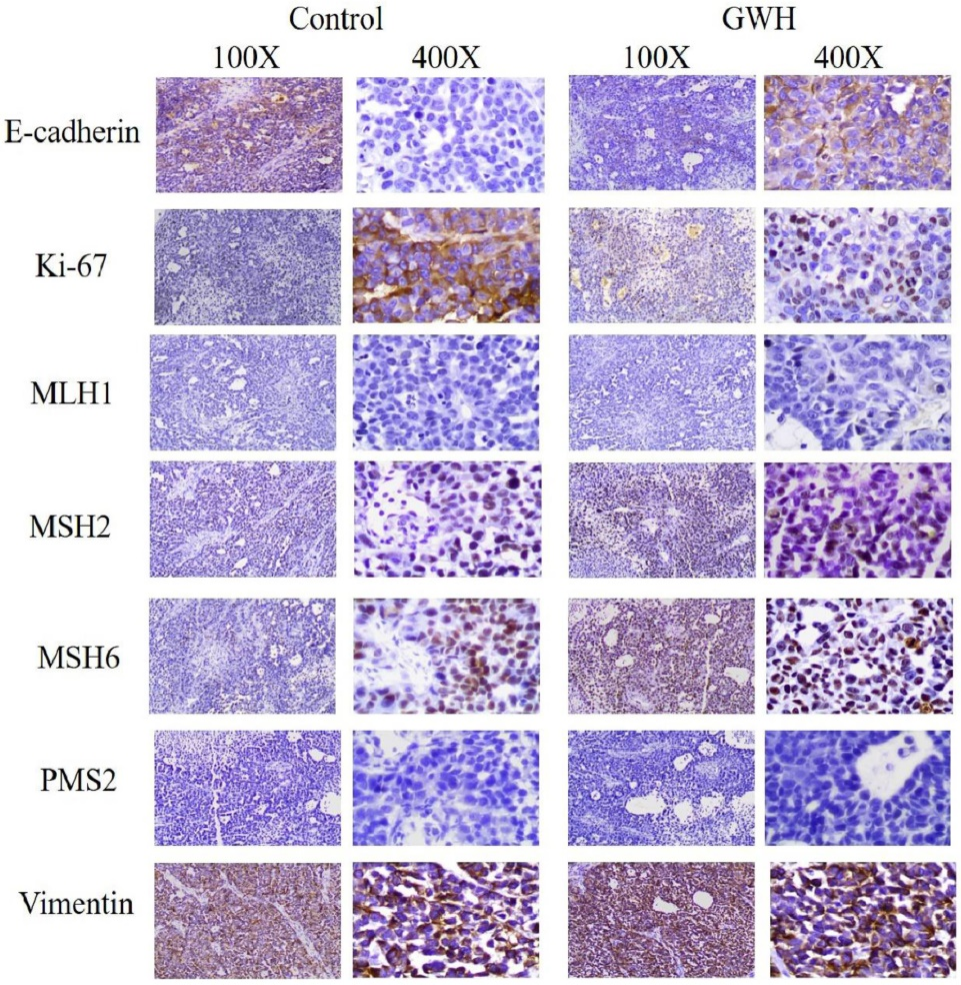
** Immunohistochemical results of tumor tissues in mice treated with GWH extracts.** After treatment with GWH extracts, the number of Ki67 nuclear staining decreased, the nuclear staining of E-cadherin deepened, Vimentin staining decreased and the number of nuclei decreased, compared with those before treatment, which indicated that GWH extracts could increase the adhesion factor E-cadherin and inhibit the expression of tumor growth factor Ki67 and Vimentin.

The immunohistochemical results showed that GWH extracted increased E-cadherin expression in SCG7901 cells and inhibited tumor growth factor Ki67and Vimentin. The above results showed that GWH extracts could inhibit gastric cancer cells' growth and invasive ability (Fig. 9).

### RNA-seq was used to detect the changes of metabolic pathway and differentially expressed genes

**Figure 10:**
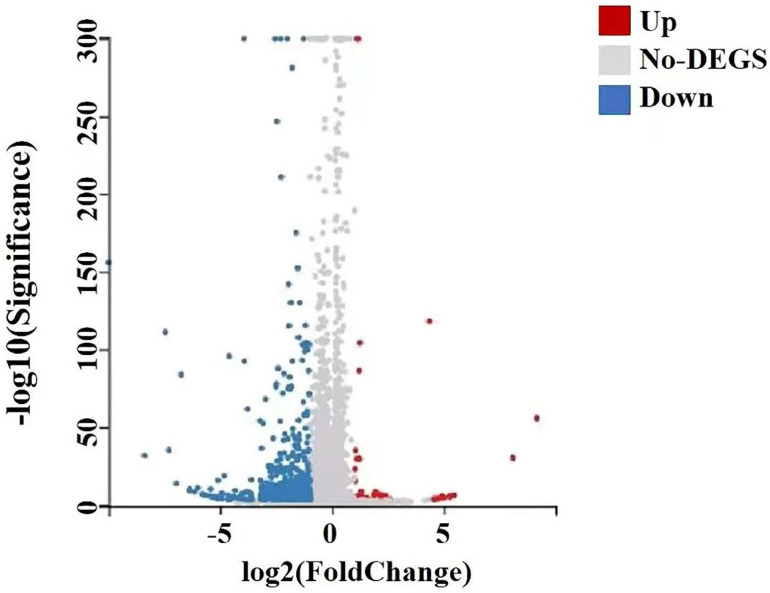
** Differential gene volcano map of transplanted tumors of gastric cancer.** The X axis represented the difference multiple value after log2 conversion, and the Y axis represented the significant value after-log10 conversion. Red represented up-regulated DEG, blue represented down-regulated DEG, gray represented non-DEG. There were 651 differentially expressed genes, of which 41 genes were up-regulated and 610 genes were down-regulated.

A total of 22,741 genes were identified by RNA-seq, of which 651 genes were differentially expressed, of which 41 genes were up-regulated and 610 genes down-regulated, and they satisfied fold-change filtering (| log2 (fold change) | > 1) and Student's t-testing (p-value < 0.05). The volcano chart presented the difference in gene expression between the experimental and control groups (Fig. 10). We clustered the FPKM values of different genes in each group, evaluated Pearson's correlation coefficient of total transcripts expression level among another group, and drew a heat map of inter-sample correlation (Fig. 10). The transcript expression level was significantly different between the experimental group and the control group, but there was no significant difference among the groups. The transcripts of these differential expressions were of great significance for the further study of the anti-tumor physiological and pathological mechanism of GWH extracts on gastric cancer cells.

### Validation for the sequencing data by qRT-PCR

**Figure 11:**
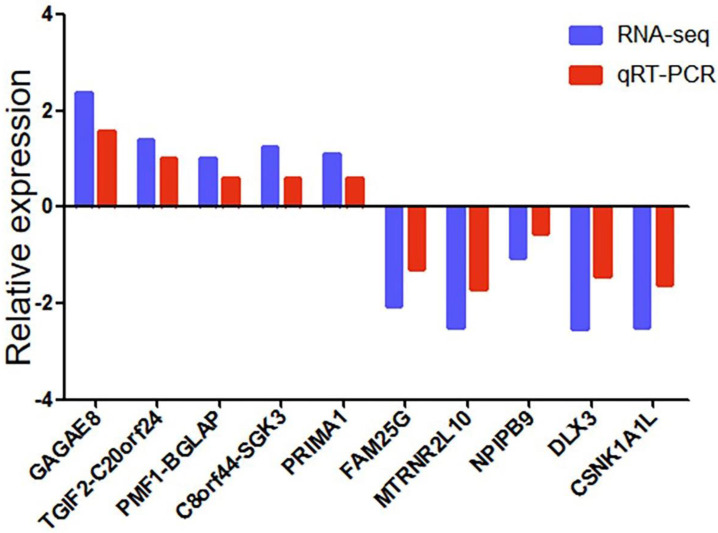
** qRT-PCR validation of ten differentially expressed genes in 10 pairs of gastric cancer samples.** Comparison of log2FC in ten differentially expressed genes between RNA-seq and qRT-PCR. Data are shown as means ± s.d. of at least three independent experiments.

We used qRT-PCR to verify the reliability of RNA-seq data. We randomly selected ten differentially expressed genes (5 up-regulated and five down-regulated). We detected whether the differentially expressed genes in SCG7901 cells treated with GWH extracts were consistent with the results of RNA-seq by qRT-PCR. The results showed that the expression levels of GAGAE8, TGIF2-C20orf24, PMF1-BGLAP, C8orf44-SGK3, PRIMA1 increased significantly. On the contrary, the levels of FAM25G, MTRNR2L10, NPIPB9, DLX3, and CSNK1A1L decreased (Fig. 11). The above data showed that the qRT-PCR results were in good agreement with our RNA-seq data, which confirmed the high reliability and validity of RNA-seq results.

### GO and KEGG analysis

**Figure 12:**
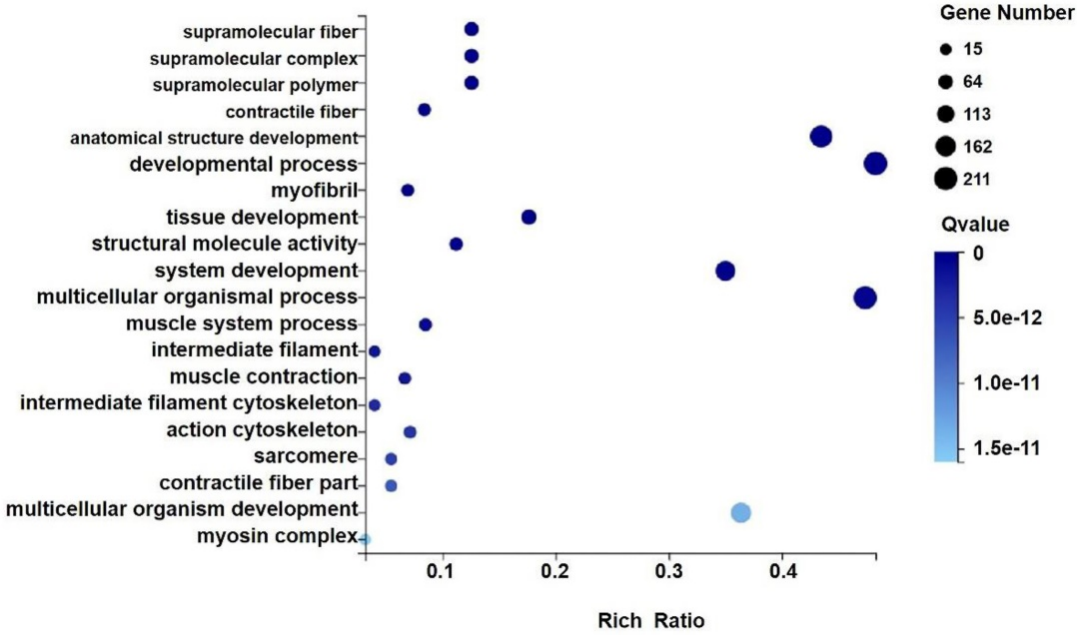
** GO analysis of differential genes expression in transplanted gastric cancer.** The X axis is the enrichment ratio [the ratio of the number of genes annotated to an entry in the selected gene set to the total number of genes annotated to the species in this species, the formula is Rich Ratio = Term Candidate Gene Num / Term Gene Num], the size of the GO Term, bubble in the Y axis indicates the number of differential genes annotated to a GO Term, the color represents the enriched Qvalue value, and the darker the color represents the smaller the Qvalue value.

**Figure 13:**
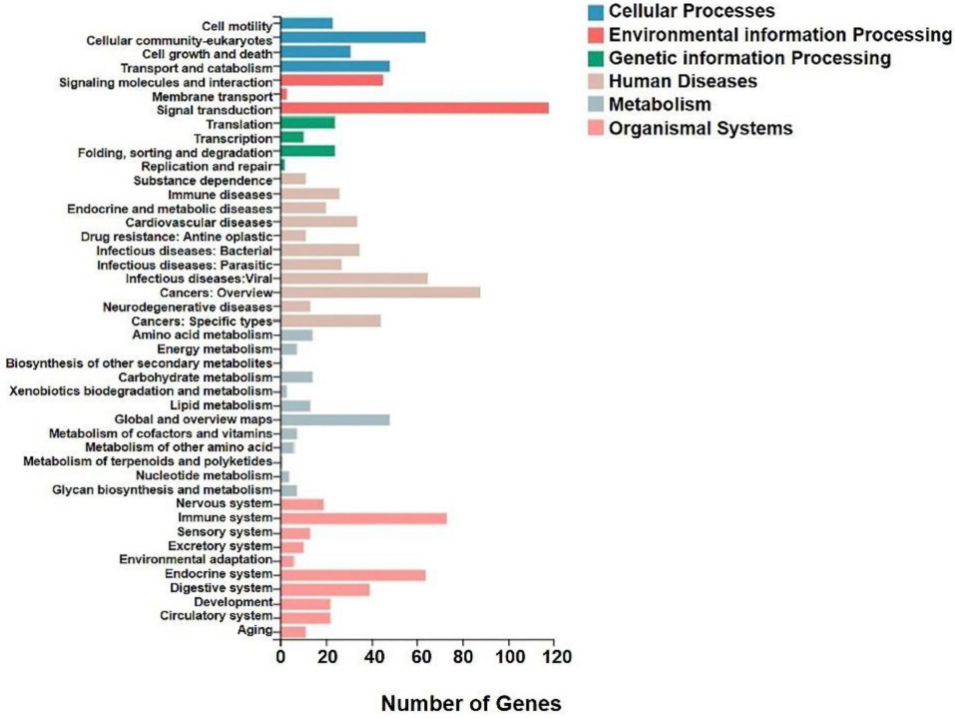
**KEGG analysis of differential genes expression in transplanted gastric cancer.** The X axis was the number of genes enriched to a certain KEGG Pathway class, and the Y axis was the KEGG Pathway category. Color indicated that the KEGG metabolic pathway was related to cellular processes, environmental information processing, genetic information processing, human diseases, metabolism, and organic systems.

We analyzed the differentially expressed genes after GWH extracts treatment by GO and KEGG enrichment analysis to predict the mechanism of anti-tumor effect of GWH extracts. Combined with the results of enrichment analysis, we screened critical regulatory pathways and critical differentially expressed genes. GO analysis covered three areas: biological process, cellular component, and molecular function. The most significantly enriched GO terms were regulation of anatomical structure development, developmental process, system development, and multicellular organismal process (Fig. 12). In the KEGG pathway analysis, the KEGG metabolic pathway involved in genes is divided into seven branches: Cellular Processes, Environmental Information Processing, Genetic Information Processing, Human Disease, Metabolism, Organismal System, Drug Development (Fig. 13). The differentially expressed genes were mainly associated with focal adhesion, ECM-receptor interaction, protein digestion and absorption, systemic lupus erythematosus, phagosome, regulation of actin cytoskeleton, etc. The above results indicated that these pathways might be involved in the GWH extracts anti-cancer effects in gastric cancer cells.

## Discussion

In recent years, traditional Chinese medicines play a significant role in anti-tumor, which has aroused widespread concern at home and abroad and have the advantages of small adverse reactions, low price, etc. Studies have shown that the green walnut husk extracts inhibit various types of cancer cells, such as esophageal cancer 26, prostate cancer 27, and breast cancer 28, and its anti-cancer effect has been proven. These studies provide a new idea for the comprehensive treatment of tumors. Our study demonstrated that the organic extracts of the green walnut husk have anti-gastric cancer activity. Our study results will help to carry forward the quintessence of Chinese culture and provide new ideas for the treatment of gastric cancer.

According to some relevant literature, we can see that the green walnut husk extracts contain a battery of bioactive compounds of different chemical types, including flavonoids, naphthoquinones, diarylheptanoids, triterpenes, phenol acid, sterol, and lipid substances. Juglone (5-hydroxy-1,4-naphthoquinone) is a chemical present, as one of the major bioactive components present in the bark of the green walnut husk, exhibits versatile bioactivities, especially anti-cancer activity 29. In the present study, the main components of organic extracts of the green walnut husk extracts were Juglone. GWH extracts suppressed the proliferation of gastric cancer cells, and increase was induced in a dose- and time-dependent manner and inhibited the migration and invasion of gastric cancer cells as well. In addition, GWH extracts inhibited the growth of mouse xenografted tumors. Therefore, this study confirmed that GWH extracts played an anti-gastric cancer effect induced by inhibiting tumor cells' proliferation and invasion.

Agents suppressing the proliferation of malignant cells by enhancing apoptosis may constitute a functional mechanistic approach to cancer chemoprevention and chemotherapy. Our results showed that GWH extracts suppressed the apoptosis of SCG7901 cells. Using AO/EB fluorescent staining assay has also observed the typical morphological changes of apoptosis, such as chromatin condensation, margination against the nuclear envelope, and apoptotic bodies' formation after GWH extracts treatment. And Flow cytometry confirmed GWH extracts induced apoptosis of SCG7901 cells. The expression of pro-and anti-apoptotic proteins was investigated in the present study, we found decreased expression of Bcl-2 protein, but increased expression of Bax, caspase3, and p53 protein Bcl-2/Bax ratio decreased. Therefore, the results demonstrated that GWH extracts played an anti-gastric cancer effect by inducing apoptosis of gastric cancer cells.

Among a variety of anti-tumor mechanisms, the most widely studied is the effect of the green walnut husk extracts on tumor cell apoptosis. Most studies have shown that it is related to the mitochondrial-dependent apoptosis pathway. For example, Green Husk of Juglans Regia L. contained bioactive compounds that could kill Pc-3 Human Prostate Cancer Cells by inducing apoptosis 27. There are two main apoptosis pathways: an extrinsic pathway (death receptor pathway) and the other is an intrinsic pathway (mitochondria pathway) 30. The mitochondrial apoptosis pathway is the most common apoptosis mechanism in cancer 31. Mitochondria are the most critical organelles that mediate intrinsic apoptosis in the body. When extracellular stimulation or intracellular signal is activated, apoptosis precursor proteins, for example, Bim, Bid, and Bad, activated apoptosis precursor proteins interact with pro-apoptotic proteins, resulting in increased expression. And then, they bind to anti-apoptotic proteins and inhibit their expression. Finally, the pro-apoptotic protein oligomeric complex is inserted into the outer membrane of the mitochondria, resulting in a decrease in the membrane potential of the mitochondria.

Furthermore, the mitochondrial membrane permeability transport pore is opened, and cytochrome C is released into the cytoplasm. Cytochrome C release into the cytoplasm can bind to Apoptotic protease activating factor-1(Apaf-1) and combine with Caspase-9 to form the structure of apoptotic bodies, which trigger downstream Caspase-9/3 signal cascades and eventually lead to cell death 32. The intrinsic pathway originates from intracellular signals and does not depend on extracellular signals, such as DNA irreversible damage, so intrinsic pathway is considered the main pathway in response to chemotherapeutic drugs and radiotherapy.

Apoptosis is strictly regulated, and some proteins are involved in the regulation of apoptosis, including p53, Bcl-2 family, and other apoptosis regulatory proteins. As we all know, the p53 gene is an essential pro-apoptotic factor and tumor suppressor, and it is also one of the most studied tumor suppressors in almost all cancers. Studies have shown that the p53 gene is mutated in more than half of human tumors. Some studies have confirmed that the p53 gene can activate both endogenous and exogenous apoptosis pathways 33. p53 is a direct transcriptional activator of the Bax gene; p53 protein interacts with Bcl-2 to enhance Bax-promoted outer-mitochondrial membrane permeabilization. The Bcl-2 family mainly regulates the pathway of mitochondrial apoptosis. In the Bcl-2 family, it is divided into pro-apoptotic proteins and anti-apoptotic proteins. As an essential anti-apoptotic protein, Bcl-2 has a particular expression in cells under normal circumstances; when the word of Bcl-2 decreases, it may promote the apoptotic response of anti-cancer drugs; while when the word of Bcl-2 increases, it may lead to resistance to chemotherapeutic drugs, radiotherapy, and other anti-tumor therapy. Bcl-2 inhibits apoptosis by negatively regulating the apoptotic activity of Bax and forming Bcl-2/Bax heterodimers. Secondly, Bcl-2 can also prevent the opening of mitochondrial membrane pores and control the opening and closing of mitochondrial membrane pores. Studies have shown that the ratio of Bcl-2/Bax can regulate the release of cytochrome C from mitochondria to the cytoplasm. Therefore, Bcl-2/Bax is considered to be a critical factor in regulating apoptosis in the endogenous mitochondrial pathway 34. Our study showed that GWH extracts could induce apoptosis in gastric cancer cells, and its mechanism was mainly related to the mitochondria pathway.

The immunohistochemical results showed that GWH extracts inhibited the expression of Ki67 and Vimentin and up-regulated the expression of E-cadherin to inhibit the growth, invasion, and migration of gastric cancer cells. E-cadherin and Vimentin play a significant role in tumor invasion and metastasis. E-cadherin is a type-I cadherin. The main role of E-cadherin is to mediate cell adhesion and maintain epithelial cell phenotype, and it plays an essential role in cell-to-cell contact inhibition of proliferation 35. E-cadherin is closely related to epithelial-mesenchymal transition (EMT), and E-cadherin is a classic tumor suppressor. E-cadherin down-regulation is considered to be a key event during EMT 36. For example, BTBD7 down-regulated E-cadherin and promoted EMT in lung cancer 37. E-cadherin is an often recurring hallmark of carcinomas, causing loss of polarity and increased proliferation, survival and invasion of epithelial cells 38. Studies have shown that the loss of E-cadherin expression can promote tumor cell invasion and metastasis, whereas increased expression of E-cadherin has been shown to reverse these phenotypes 39. Therefore, E-cadherin loss being required for cancer cells to undergo EMT in gaining invasiveness. E-cadherin signaling mediated adherens junctions (AJs) are hubs of intracellular signaling that regulate cancer cells proliferation, survival, invasion, and migration. E-cadherin is involved in several oncogenic signaling pathways, including EGF/ EGFR and Wnt/β-catenin^40^, MAPK 41, PI3K/AKT and Rho GTPase 42 and the HIPPO signaling pathway 42, 43, whereby it plays a role in many tumors, including gastric cancer. Vimentin is one of the most widely expressed and highly conserved proteins of the type III intermediate filaments (IFs) protein family. The main roles of Vimentin include affecting cell structure, integrity, stress reaction, and may influence cell signaling. The overexpression of Vimentin in tumors was closely related to accelerated growth, invasion, and poor prognosis, and Vimentin also has been recognized as a marker for epithelial-mesenchymal transition (EMT) 44. For example, the study found that the coexpression of FOXK1 and Vimentin enhanced cell metastasis through the induction of EMT in gastric cancer cells 45. There was overexpression of Vimentin in many kinds of cancer cells. Recent data suggested that vimentin intermediate filaments played an essential role in cancer cell migration, cell adhesion structures, and metastasis formation 46. Our results indicated that Vimentin and E-cadherin might be potential targets for tumor therapy.

High-throughput sequencing (NGS) has been increasingly used in cancer genomics research. A growing number of studies have demonstrated that the aberrant expressions of genes contribute to cell proliferation, metastasis, and drug resistance in cancer. In the study, we used RNA-seq to detect differential genes in tumor tissues and the metabolic pathways and signal pathways that differentially expressed genes are mainly involved in. As a result, we identified 41 up-regulated genes and 610 down-regulated genes. Ten genes were chosen for qRT-PCR validation in experimental group tissues. The results were highly consistent with the RNA-seq data, confirming the reliability of the RNA-seq results. To explore the potential functions of genes differentially expressed in gastric cancer tissues, we performed GO and KEGG pathway analyses of significantly dysregulated genes. In the GO analysis, the most significant biological processes were regulation of anatomical structure development, developmental process, system development and multicellular organismal process. Moreover, the KEGG pathway analysis provided a deep insight of the mechanism of the green walnut husk extracts in gastric cancer cells. The results showed that the potential signaling pathways such as focal adhesion, ECM-receptor interaction, protein digestion and absorption, systemic lupus erythematosus, phagosome, regulation of actin cytoskeleton might be involved. In summary, we found that the green walnut husk extracts can produce significant changes in cell movement ability, cell growth and death-related genes, which directly affect the survival, invasion and metastasis of tumor cells. In addition, genes related to signal molecular interaction and signal transduction, regulatory amino acids, lipids, carbohydrates and energy metabolism were also differentially expressed, indicating that the green walnut husk extracts affect the metabolism and related biological functions of tumor cells by regulating complex signal pathways. Our data will provide new insight into the molecular mechanism of the green walnut husk extracts toward cancer cells and facilitate understanding the green walnut husk extracts in gastric cancer.

## Conclusions

In summary, our study evaluated and validated the efficacy of GWH extracts in the treatment of gastric cancer *in vitro* and *in vivo*. Rna-seq detected the expression of differential genes in tumor tissue. The results showed that the green walnut husk extracts had an anti-gastric cancer effect mainly by inhibiting the invasion of gastric cancer cells and inducing its apoptosis. RNA-seq detected differentially expressed genes, combined with GO and KEGG analysis, we analyzed the differentially expressed genes, revealed the possible differential functions of differentially expressed genes, and the main metabolic pathways and signal pathways involved in differentially expressed genes, and screened the key regulatory pathways and key differentially expressed genes. Our results will provide new insights into the mechanism of the green walnut husk extracts on cancer cells, and the green walnut husk extracts will be a potentially safe and effective drug to inhibit the growth and invasion of gastric cancer cells.

## Deficiency

This is the first time to study the effect of the green walnut husk extracts on gastric cancer cells. In the present study, first, *in vivo*, we treated the gastric cancer cells with the green walnut husk extracts. Then the proliferation, apoptosis, migration, and invasion of the gastric cancer cells were detected by using the CCK-8, flow cytometry, Western blot, and other assays. Moreover, the growth of gastric cancer cells was assessed by using orthotopic xenograft models. Finally, the Gene expression profile of gastric cancer treated with the green walnut husk extracts was assessed using RNA-seq. According to the results, the green walnut husk extracts inhibited the proliferation, migration, and invasion of gastric cancer cells and induced apoptosis. These differentially expressed genes may be related to the mechanism of the anti-gastric cancer effect of the green walnut husk extracts. In addition, Ali A Alshatwi et al. reported that green husk extracts suppressed human prostate cancer cell proliferation and induced its apoptosis by modulating the expression of apoptosis-related genes 27, which is consistent with our results.

There are still many deficiencies in this study. Our study only used one cell line, which lacked of normal and other cancer cells as controls. And we failed to use any of the commonly used validation methods. Our article only preliminarily discussed the role of the green walnut husk extracts in gastric cancer and its potential mechanism. The results could provide new insight into the mechanism of the green walnut husk extracts on cancer cells, and it is further proved that the green walnut husk extracts may be a potential drug for gastric cancer. However, we have not thoroughly studied the specific mechanism of the green walnut husk extracts, including the central metabolic pathways and signal pathways, which need further clarification in future studies.

## Abbreviations

AO/EB: acridine orange/ethidium bromide
AJs: adherens junctions
Apaf-1: apoptotic protease activating factor-1
BCL-2: B-cell lymphoma-2
CCK-8: cell counting kit-8
EMT: epithelial-mesenchymal transition
MDSCs: Myeloid-derived suppressor cells
FCM: flow cytometry
GC: gastric cancer
GO: Gene Ontology
GWH: the green walnut husk
KEGG: Kyoto Encyclopedia of Genes and Genomes
MET: mesenchymal-epithelial transition
